# Effect of *Cymbopogon martini* (Roxb.) Will.Watson essential oil on antioxidant activity, immune and intestinal barrier-related function, and gut microbiota in pigeons infected by *Candida albicans*

**DOI:** 10.3389/fphar.2024.1380277

**Published:** 2024-04-02

**Authors:** Ting Huang, Zheng-Yue Zhang, Zhi-Lin Qiu, Lin Li, Xian-Xi Liu, Lei Wang, Zi-Ying Wang, Zhi-Peng Li, Geng-Sheng Xiao, Wei Wang

**Affiliations:** ^1^ Zhongkai University of Agriculture and Engineering, Guangzhou, China; ^2^ Meizhou Jinlv Modern Agriculture Development Co., Ltd., Meizhou, China; ^3^ Guangdong Baoning Agriculture and Animal Husbandry Technology Co., Ltd., Meizhou, China

**Keywords:** *Candida* albicans, Cymbopogon martini essential oil, pigeon, antioxidant activity, immune response, intestinal barrier function, intestinal microbiota

## Abstract

Essential oils are potential alternatives to antibiotics for preventing *Candida* albicans (C. albicans) infection which is responsible for economic losses in the pigeon industry. *Cymbopogon martini* essential oil (EO) can inhibit pathogens, particularly fungal pathogens but its potential beneficial effects on *C. albicans*-infected pigeons remain unclear. Therefore, we investigated the impact of *C*. *martini* EO on antioxidant activity, immune response, intestinal barrier function, and intestinal microbiota in *C. albicans*-infected pigeons. The pigeons were divided into four groups as follows: (1) NC group: *C. albicans* uninfected/*C*. *martini* EO untreated group; (2) PC group: *C. albicans* infected/*C*. *martini* EO untreated group; (3) LPA group: *C. albicans* infected/1% *C*. *martini* EO treated group; and (4) HPA group: *C. albicans* infected/2% *C*. *martini* EO treated group. The pigeons were infected with *C. albicans* from day of age 35 to 41 and treated with *C*. *martini* EO from day of age 42 to 44, with samples collected on day of age 45 for analysis. The results demonstrated that *C*. *martini* EO prevented the reduction in the antioxidant enzymes SOD and GSH-Px causes by *C. albicans* challenge in pigeons. Furthermore, *C*. *martini* EO could decrease the relative expression of *IL-1β*, *TGF-β*, and *IL-8* in the ileum, as well as *IL-1β* and *IL-8* in the crop, while increasing the relative expression of *Claudin-1* in the ileum and the crop and *Occludin* in the ileum in infected pigeons. Although the gut microbiota composition was not significantly affected by *C*. *martini* EO, 2% *C*. *martini* EO increased the abundance of *Alistipes* and *Pedobacter*. In conclusion, the application of 2% *C*. *martini* EO not only enhanced the level of antioxidant activity and the expression of genes related to intestinal barrier function but also inhibited inflammatory genes in *C. albicans*-infected pigeons and increased the abundance of gut bacteria that are resistant to *C. albicans*.

## Introduction

Pigeon candidiasis, also known as avian candidiasis or thrush, is a fungal infection caused by *Candida* species, most commonly *Candida albicans* (*C. albicans*). (Rosario Medina et al., 2017). It affects various parts of the pigeon including the crop, mouth, esophagus, and respiratory system (Pollock, 2003). The symptoms of pigeon candidiasis can vary depending on the affected area but commonly include the presence of white to yellowish plaques or lesions in the mouth, throat, or crop (Talazadeh et al., 2022). Conventional treatment options for pigeon candidiasis involve the use of antifungal medications such as fluconazole, itraconazole, and ketoconazole (Martin, 1999). However, many countries have restricted the use of antibiotics owing to the emergence of resistant strains, disruption of the gut microbiota, and residual environmental contamination (Lange et al., 2016; Tang et al., 2017; Hanna et al., 2018; Magalhães Pinto et al., 2019).

Essential oils are potential alternatives to antibiotics, with essential oils such as tea tree, lavender, eucalyptus, and peppermint essential oil exhibiting broad-spectrum antimicrobial activity against a range of bacteria, fungi, and viruses. (Bassolé and Juliani, 2012; Murbach Teles Andrade et al., 2014; Chouhan et al., 2017). It is believed that essential oils disrupt the cellular structure of pathogens, inhibit their growth, and promote the removal of pathogenic microorganisms in the infected animal (Szczepanski and Lipski, 2014; Omonijo et al., 2018; Andrade-Ochoa et al., 2021). Furthermore, essential oils are effective against various drug-resistant strains such as methicillin-resistant *Staphylococcus aureus* (MRSA) and multidrug-resistant *Escherichia coli* (MDR *Escherichia coli*) (Chao et al., 2008; Yap et al., 2015). Moreover, essential oils have advantages over other antibiotic substitutes. They are natural substances extracted from plants that retain the original properties and natural state of the plant relative to some antibiotic substitutes. They act through a variety of mechanisms such as inhibiting microbial growth, damaging cell membranes, and affecting microbial metabolism. This multiple mechanism of action may reduce the development of microbial resistance to essential oils compared to some antibiotic substitutes with a single mechanism of action. *Cymbopogon martini* (Roxb.) Will. Watson [Poaceae] is commonly called as palmarosa. *Cymbopogon martini* essential oil (EO) has also been shown to possess antibacterial activity against a variety of fungi, such as *C. albicans* (Singh et al., 2018). Previously, we evaluated the minimal inhibitory concentration (MIC) of several essential oils agains*t C. albicans* showing that *C*. *martini* EO was more effective against *C. albicans in vitro* than other essential oils.

However, the effect of *C*. *martini* EO on the *C. albicans* infected pigeons remains to be elucidated. Therefore, this study was designed to investigate the effects of *C*. *martini* EO on antioxidant activity, intestinal barrier function, immune-related genes, and gut microbiota in *C. albicans*-infected pigeons. This study provides a scientific basis for developing environmentally friendly prevention strategies for *C. albicans*-infected pigeons to improve their health status and promote the sustainable development of the breeding industry.

## Material and methods

### Ethics statement

This study was carried out in accordance with the principles of the Basel Declaration and recommendations of the Institutional Animal Care and Use Committee guidelines, Zhongkai University of Agriculture and Engineering.

### Bacterial strains and *C. martini* EO

The pathogenic *C. albicans* was isolated from the intestines of pigeons infected with *C. albicans* in Guangdong Province, China. The strain was stored at −80°C in fluid thioglycollate broth (FT, Beckson, Dickinson, and Company) supplemented with 30% glycerol. *Cymbopogon martini* EO were obtain from Guangzhou Rui Cheng Biotechnology Co., Ltd. (Guangzhou, China, batch no: 6–296695, sell by date:22/11/2025). *Cymbopogon martini* EO is obtained by distillation of the whole plant (the main chemical composition: myrcene 0.2%, *cis*-*β*-Ocimene 0.3%, *trans-β*-Ocimene 1.3%, linalool 2.2%, neral 0.1%, geraniol 67.7%, geranial 0.1%, geranylacetate 24.6%, caryophyllene 0.5%, gerany isobutyrate 0.1%, farnesol 0.9%). *Cymbopogon martini* EO were dissolved in soybean oil with 1% (v/v) and 2% (v/v) concentration and then stored at 4°C until use.

### Animal trial design and sample collection

A total of eighty 30-day-old pigeons (negative for *C. albicans*) were purchased from a hatchery in Guangzhou city, Guangdong Province, China. They were randomly allocated to one of four experimental groups. Food and water were provided *ad libitum*, and the pigeons were maintained with 12-h light/dark cycles. The animal trials were conducted at the pigeon house located at Zhongkai University of Agriculture and Engineering. Four groups (20 pigeons per group) were designed: (1) NC group: *C. albicans* uninfected/*C*. *martini* EO untreated group; (2) PC group: *C. albicans* infected/*C*. *martini* EO untreated group; (3) LPA group: *C. albicans* infected/1% *C*. *martini* EO treated group; and (4) HPA group: *C. albicans* infected/2% *C*. *martini* EO treated group.

All pigeons were fed an antibiotic-free basic diet for pigeons (Supplementary Table S1). Groups PC, LPA, and HPA were orally gavaged once per day with an actively growing 200 μL concentration of 2 × 10^7^ cfu/ml *C albicans* from 35 to 41 days of age. Group NC orally received sterilized normal saline in the same volume. Following infection, the pigeons gradually displayed symptoms such as mental depression, indigestion, and reduced ingestion. From 39 to 41 days of age, two pigeons were euthanized daily, and their crops were dissected to observe the status of *C. albicans* infection. The white pseudofilm in the crop was swabbed with a cotton swab and identified as *C. albicans* to confirm the success of the inoculation. It was observed that all inoculated groups were successfully infected with *C. albicans*. Meanwhile, Groups LPA and HPA were administered 200 μL of 1% and 2% (according to the previous studies (Zeng et al., 2011)) *C*. *martini* EO once per day from 42–44 days of age, respectively. Groups NC and PC were orally given soybean oil in the same volume. None of the pigeons died during the experiment. This animal experimental design is shown in Table 1.

**TABLE 1:** Experiment design with pigeons.

Experimental treatment	Components received by pigeons		
	*C*. *albicans* infection	1% *C. martini EO*	2% *C. martini EO*
NC-Negative control group	-	-	-
PC-Positive control group	+	-	-
LPA-low dose *C. martini EO*	+	+	-
HPA-high dose *C. martini EO*	+	-	+

At 45 days of age, blood samples were collected from seven pigeons in each group via the brachial wing vein using sterile syringes. The collected blood was centrifuged at 5000 *g* for 15 min at room temperature. The isolated serum was stored at −20°C for subsequent antioxidant activity testing. Subsequently, the pigeons were euthanized by cervical dislocation after blood collection. The jejunum-ileum content was extruded manually for subsequent DNA extraction. All intestinal content samples were immediately placed into sterile plastic tubes using alcohol-sterilized spatulas, snap-frozen in liquid nitrogen, and stored at −80°C until analysis. The ileum and crop tissues were dissected out. After slitting the tissue lengthwise and gently rinsing with ice-cold 1 × PBS, the collected tissues were immediately frozen in liquid nitrogen for RNA isolation.

### Serum antioxidant activity test

The serum concentration of total antioxidant capacity (T-AOC), the superoxide dismutase (SOD), glutathione peroxidase (GSH-Px) and catalase (CAT) were determined according to the manufacturer’s instructions accompanying the assay kit, which involved a T-AOC, SOD, GSH-Px and CAT assay kit, (Nanjing Jiancheng Bioengineering Institute).

### RNA isolation and RT-qPCR of the immune and intestinal barrier-related genes

Using the EZNA^®^ Total RNA Isolation Kit (Omega Bio-Tek), RNA was isolate from ileal and crop tissue according to the manufacturer’s protocol. RNA was eluted in DEPC-treated water and stored at −80°C. Total RNA was quantified using a NanoDrop^®^ ND-2000 UV spectrophotometer (NanoDrop Technologies, Wilmington, DE, United States). Reverse transcription was performed with M-MLV Frist Strand cDNA Synthesis Kit (Omega Bio-Tek), following the manufacturer’s protocol. Quantitative PCR reaction was performed with cDNA temple in triplicate using SYBR Premix Ex Taq (TAKARA Bio, Otsu, Japan). The target genes values were normalized to the housekeeping gene encoding β-actin. The relative mRNA level of each target gene was calculated based on the expression of the β-actin using 2 ^−ΔCt^ method. The primers used for qPCR of interleukin (IL)-1β, transforming growth factor (TGF) -β, IL-8, claudin-1, occludin, Zona occludens (ZO)-1and β-actin are showed in Supplementary Table S2.

### DNA extraction and 16S rRNA gene sequencing

DNA from pigeons jejunum–ileum content contents were extracted using QIAamp Fast DNA Stool Mini Kit (Qiagen, Valencia, CA, United States) according to the manufacturer’s instructions. Total DNA was quantified using a NanoDrop^®^ ND-2000 UV spectrophotometer (NanoDrop Technologies, Wilmington, DE, United States). The instrument measures absorbance at 260 nm (A260) to quantify DNA in samples, at 280 nm (A280) to verify protein contamination and at 230 nm (A230) for determining contamination by phenol. Only DNA samples with A260/A280 ratio as 1.7 and A260/A230 > 1.8 were used for further analysis. The extracts were stored at −20°C until use.

The V3-V4 hypervariable regions of 16S rDNA were PCR amplified from microbial DNA. The gene-specific sequences for the 16S V3 and V4 region were performed by using primers 341F 5′-CCTACGGGNGGCWGCAG-3′and 805R 5′-GACTACHVGGGTATCTAATCC-3’. The PCR conditions were as follows: one pre-denaturation cycle at 94°C for 4 min, 25 cycles of denaturation at 94°C for 30 s, annealing at 55°C for 45 s, and elongation at 72°C for 30 s, and one post-elongation cycle at 72°C for 5 min. The PCR amplicons were separated on 0.8% agarose gels and then extracted using QIAEX II gel extraction kit (Qiagen) according to the handbook. Only PCR products without primer dimers and contaminant bands were used for sequencing. Amplicons were purified using AMPure X using the manufacturer’s instructions (Beckman Coulter, Mississauga, ON, Canada). Bar-coded V3 and V4 amplicons were sequenced using the 2 × 250 paired-end method by Illumina NovaSeq with a seven-cycle index read. Sequences processing was performed using QIIME to get clean data. Sequences with an average Phred (Q) score lower than 30, with ambiguous bases or homopolymer runs exceeding six bp, primer mismatches or sequence lengths shorter than 100 bp were removed. The consensus sequence was generated by FLASH (Fast length Adjustment of Short reads, v1.2.11) as following: only sequences with an overlap longer than 10 bp and without any mismatches were assembled according to their overlap sequences. Reads that could not be assembled were discarded. Barcode and sequencing primers were trimmed from the assembled sequence. The high quality paired-end reads were combined to tags based on overlap. Sequences analysis were performed by Uparse software. Sequences with 97% similarity were assigned to the same OTUs. OTU representative sequences were taxonomically classified using the Silva Database based on Mothur algorithm to annotate taxonomic information.

### Statistical analysis

The relative expression of mRNA and the alpha diversity indices of Shannon, Simpson and ACE among the four groups were tested using ANOVA with a *p*-value of <0.05 considered to represent significance. Genus and species abundance were compared using Kruskal–Wallis test with Benjamini–Hochberg *p*-value correction.

## Results

### Serum antioxidant activity

**FIGURE 1:**
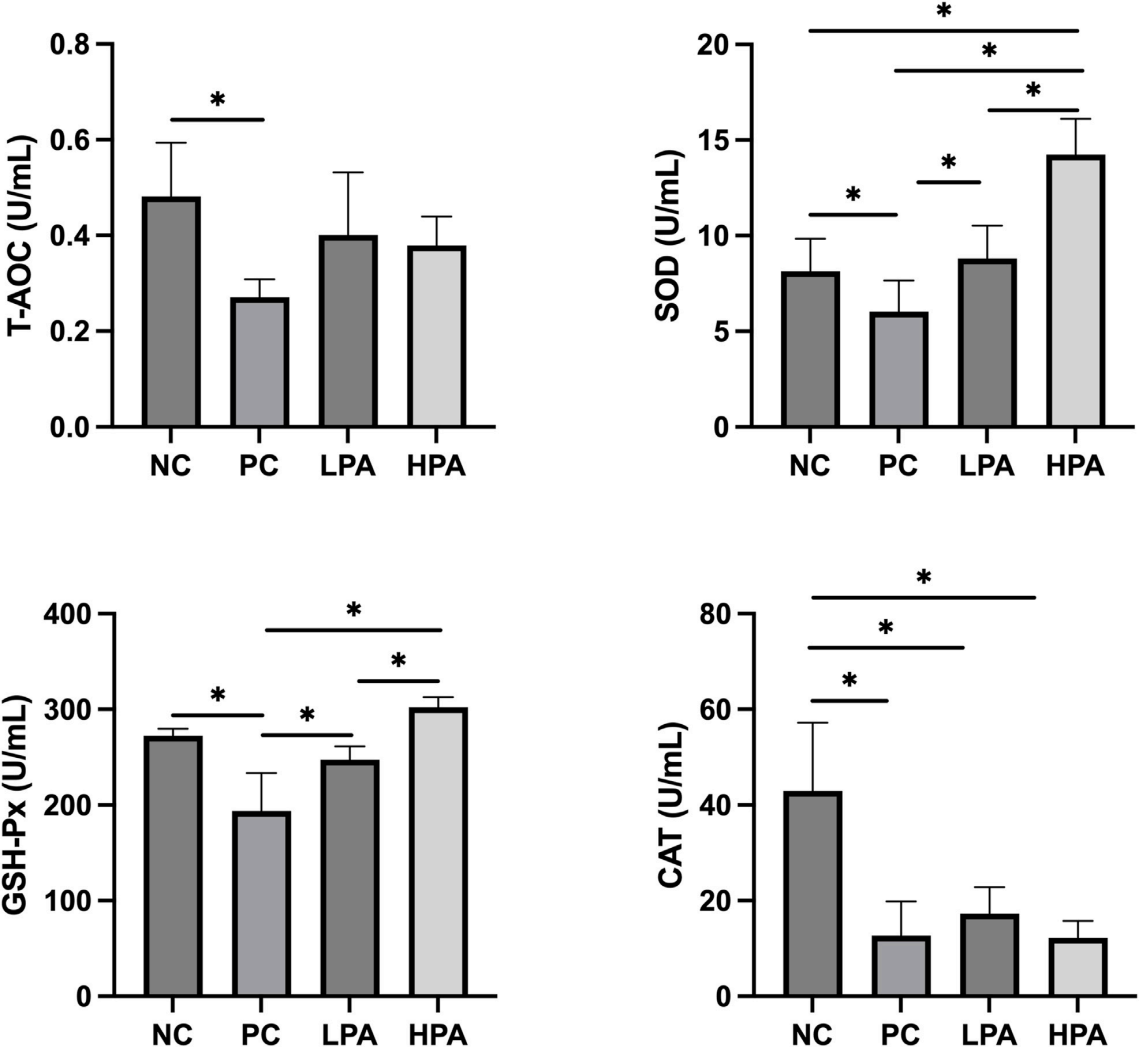
Effect of *C. martini* EO on serum antioxidant levels in *C. albicans* infected pigeons NC: *C. albicans* uninfected/*C*. *martini* EO untreated group, PC: *C. albicans* infected/*C. martini* EO untreated group, LPA: *C. albicans* infected/1% *C. martini* EO treated group, HPA: *C. albicans* infected/2% *C. martini* EO treated group. *p < 0.05.

Figure 1 shows the effects of *C. albicans* and *C*. *martini* EO on antioxidant levels, including superoxide dismutase (SOD), total antioxidant capacity (T-AOC), catalase (CAT), and glutathione peroxidase (GSH-Px). The T-AOC, SOD, GSH-Px, and CAT levels in the PC group were significantly lower than those in the NC group (*p* < 0.05). Notably, the concentrations of SOD and GSH-Px increased significantly in the LPA and HPA groups compared to the PC group (*p* < 0.05). Furthermore, the levels of SOD and GSH-Px in the HPA group were significantly higher than those in the LPA group (*p* < 0.05).

### Expression of immune and intestinal mucosa barrier-related genes

**FIGURE 2:**
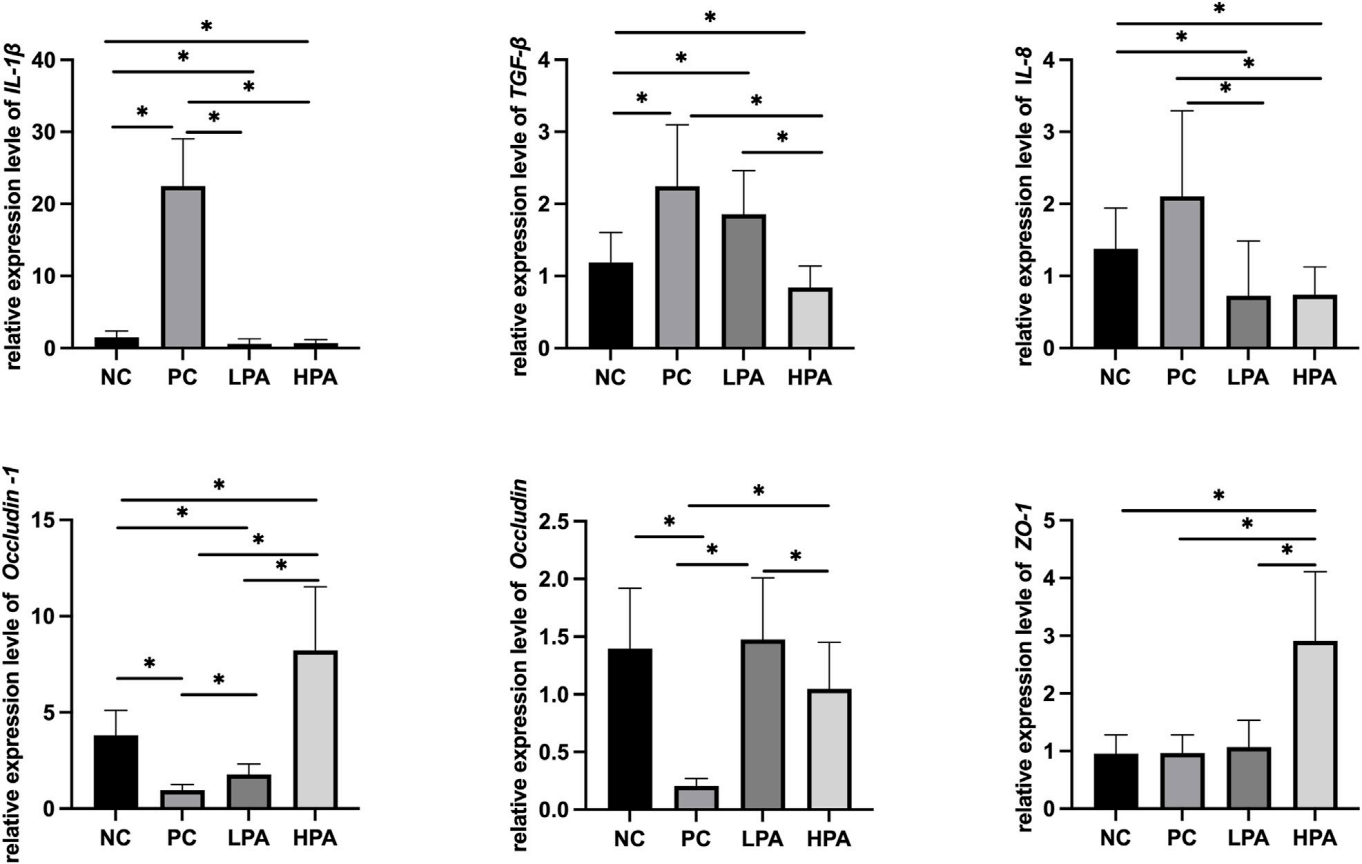
mRNA expression level in ileum. Relative gene expression presented as log^10^, ∗*p* < 0.05, ANOVA.

**FIGURE 3:**
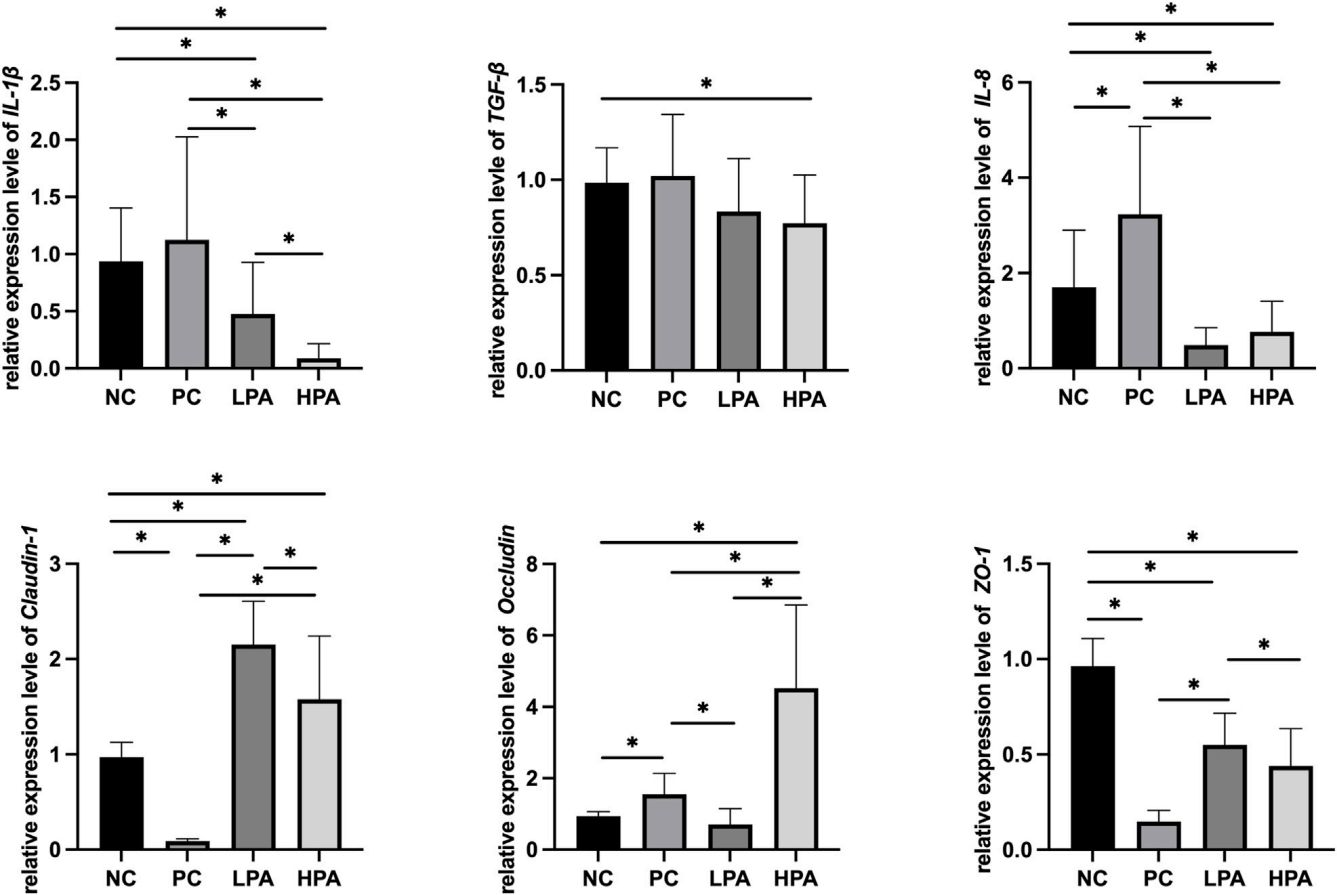
Relative change in gene expression in crop tissue Relative gene expression represented as log^10^, ∗*p* < 0.05, ANOVA.

Figure 2 and Figure 3 show the expression of immune and intestinal barrier-related genes observed in the four different groups Regarding the expression of immune genes, the relative expression of *IL-1*β and *TGF-β* in the ileum, and *IL-8* in the crop, was significantly higher in the PC group compared to the NC group (*p* < 0.05). Additionally, the relative expression of *IL-1*β, *TGF-β*, and *IL-8* in the ileum, as well as *IL-1*β and *IL-8* in the crop, was lower in the LPA and HPA groups than in the PC group (*p* < 0.05). Regarding barrier-related genes, the relative expression of *Claudin-1* and *Occludin* in the ileum, and *Claudin-1* and *Z O -1* in the crop, was significantly lower in the PC group compared to the NC group (*p* < 0.05). However, the relative expression of *Claudin-1* and *Occludin* in the ileum, as well as *Claudin-1* in the crop, significantly increased in the LPA and HPA groups compared to the PC group (*p* < 0.05). Furthermore, the relative expression of *Z O -1* in the ileum, and *Occludin* in the crop, was higher in the HPA group than in the PC group (*p* < 0.05). Finally, the relative expression of *Z O -1* in the crop was significantly higher in the LPA group than in the PC group (*p* < 0.05).

### Intestinal bacterial structure analysis

**FIGURE 4:**
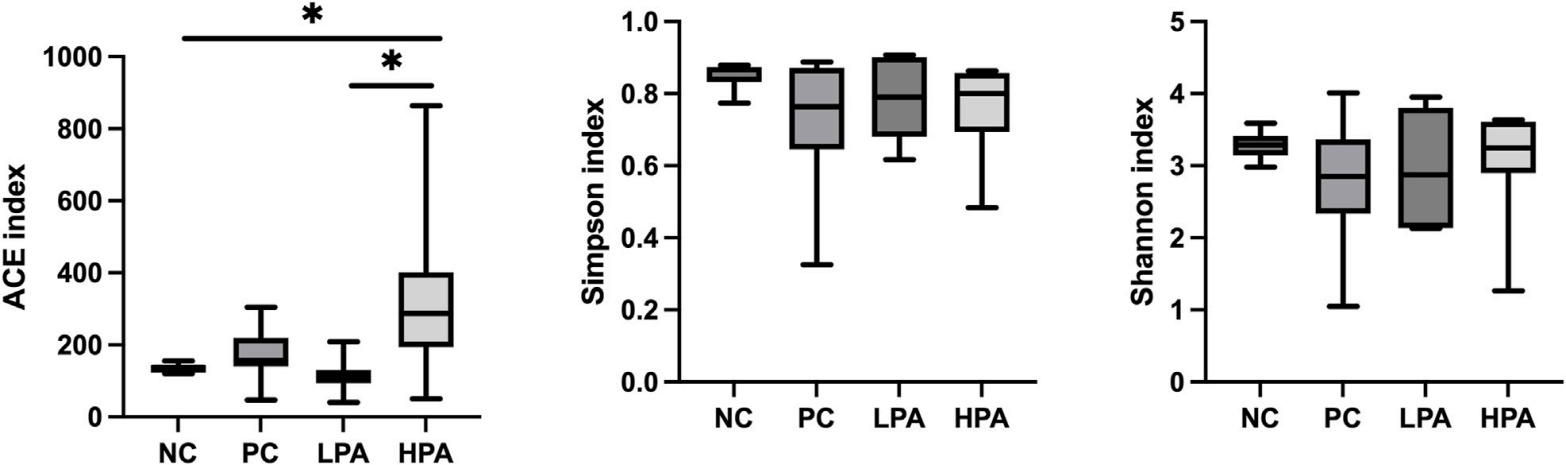
Alpha diversity of the intestinal bacterial community in the four pigeon groups. ∗*p* < 0.05, ANOVA.

**FIGURE 5:**
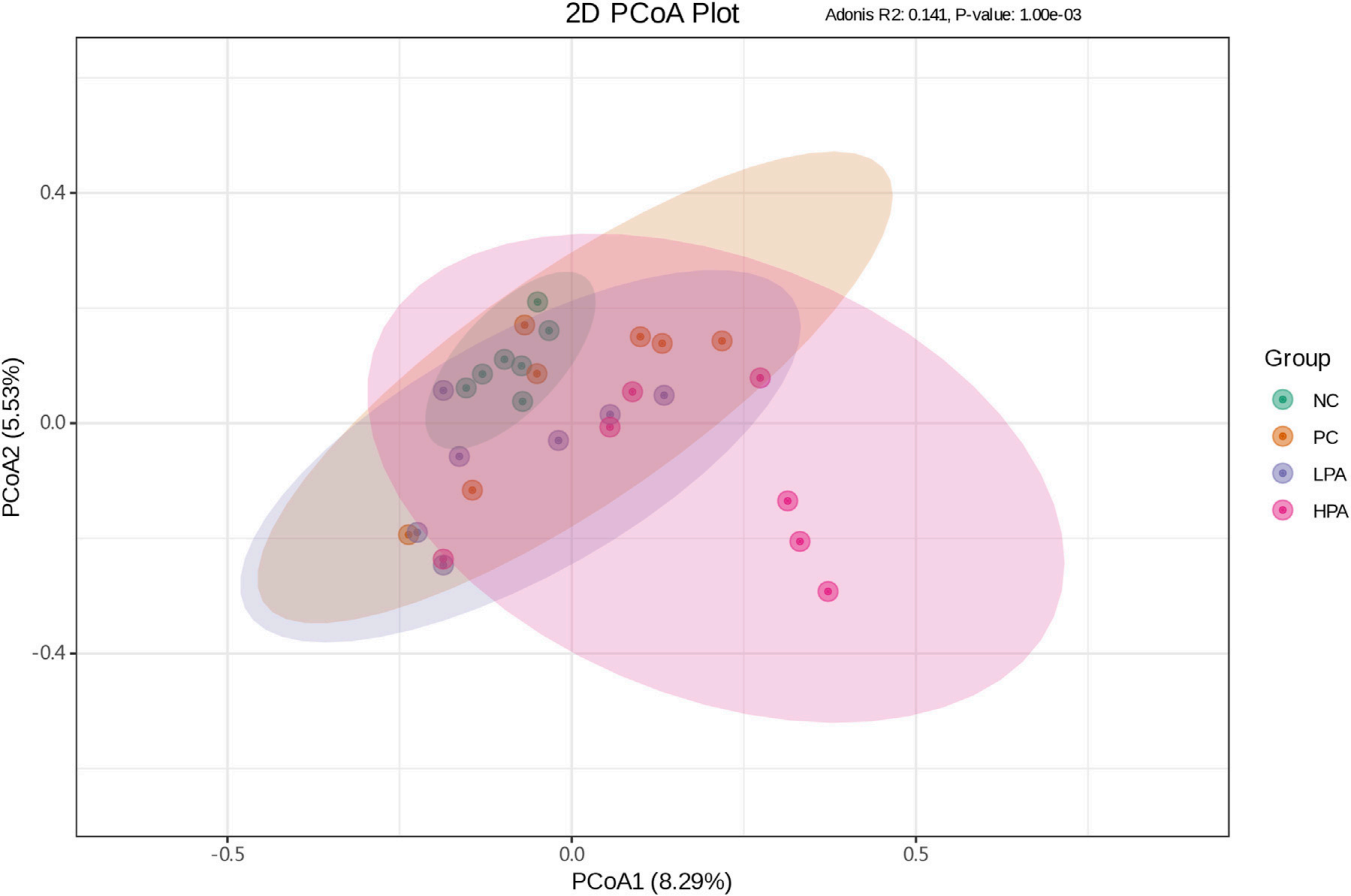
Principal coordinate analysis plot of binary-Jaccard indices for the gut microbiota of the four pigeon groups. The x-coordinate represents one principal component, the y-coordinate represents another principal component, and the percentage represents the contribution of the principal component to the sample difference. Each point in the diagram represents one sample, and samples from the same group are represented in the same color. The green, orange, purple and pink points indicate groups NC, PC, LPA and HPA respectively.

**FIGURE 6:**
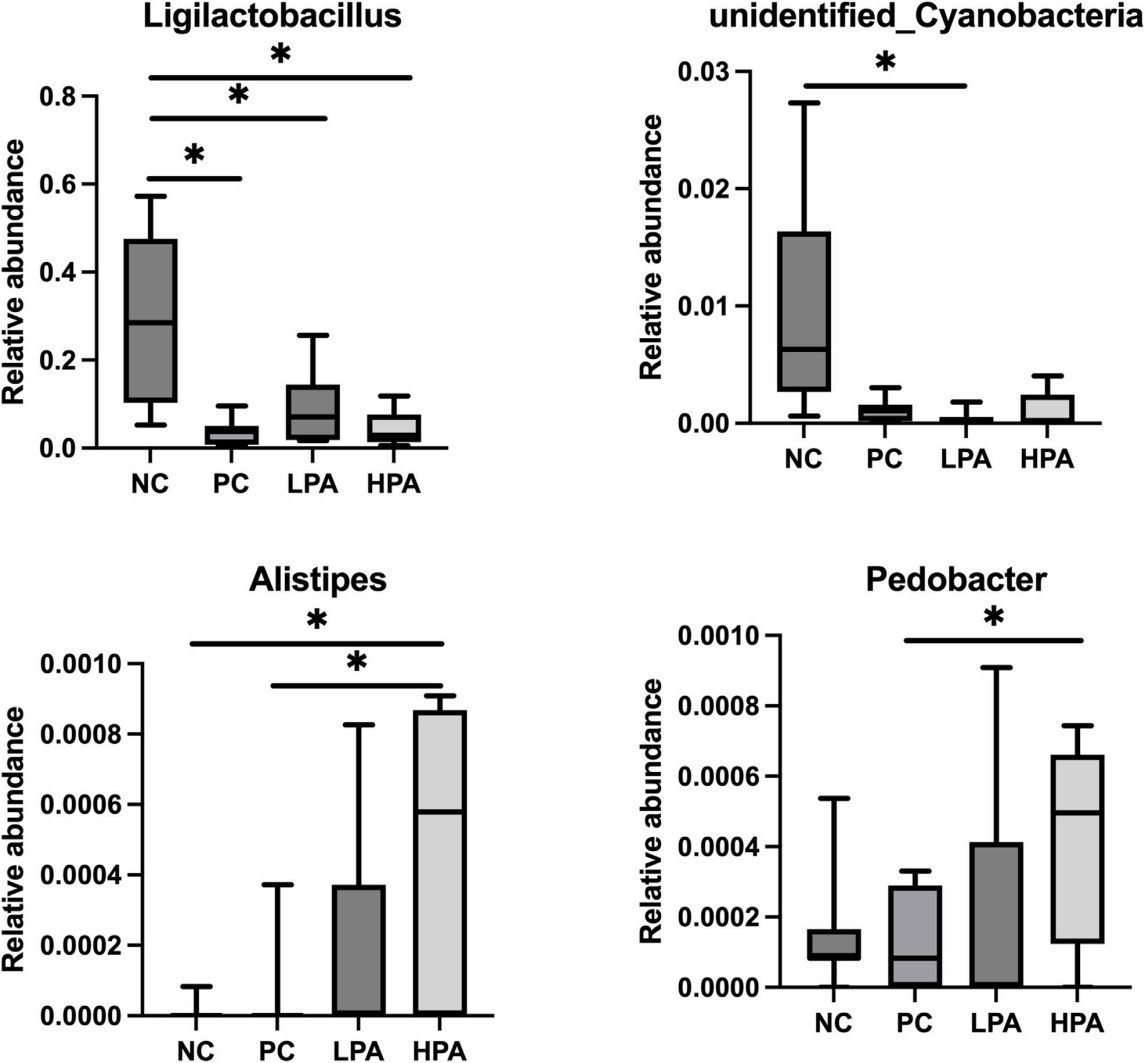
Comparison of gut bacterial composition at the genus level using relative abundance. ∗*p* < 0.05, Kruskal–Wallis test.

**FIGURE 7:**
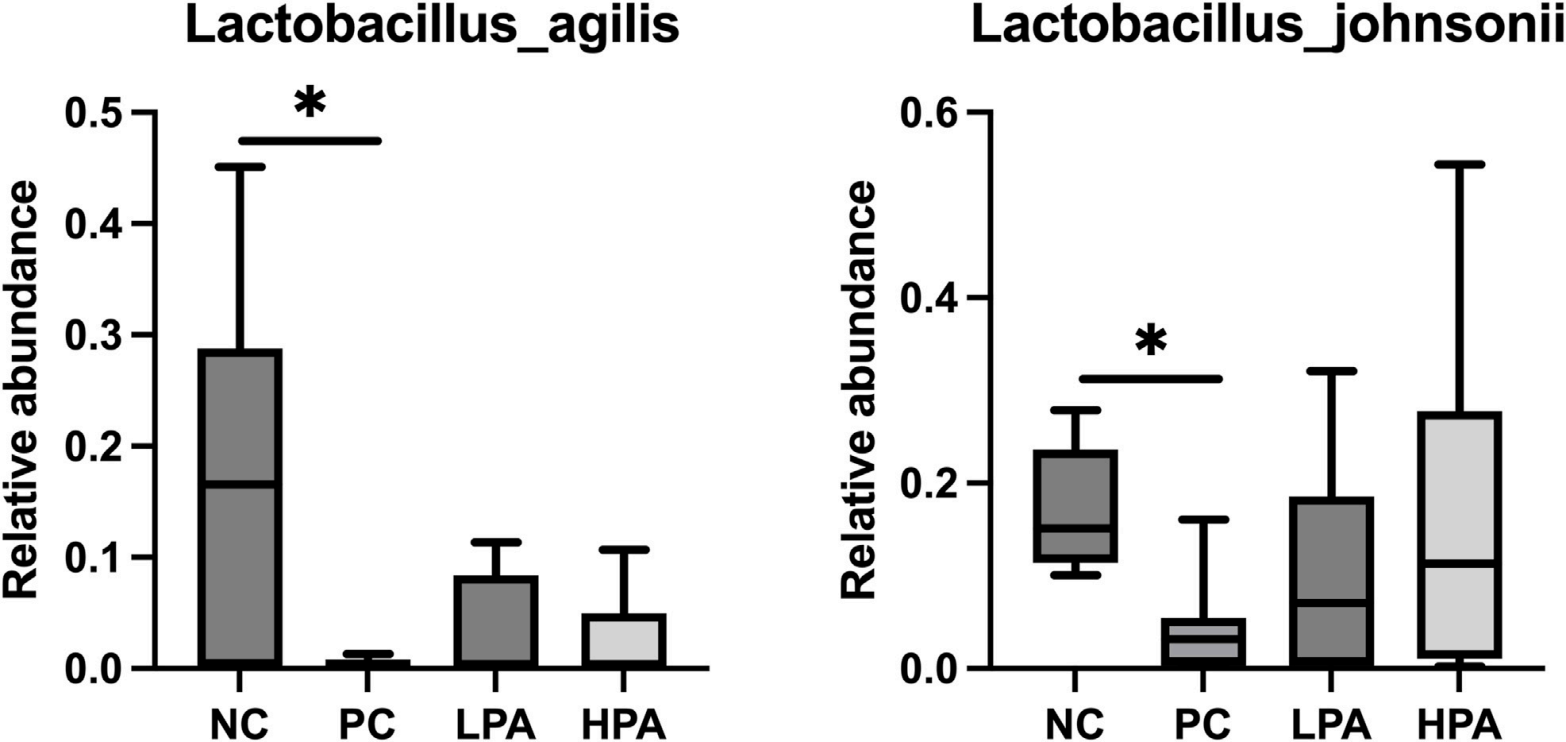
Comparison of gut bacterial composition at the species level using relative abundance.∗*p* < 0.05, Kruskal–Wallis test.

The alpha diversity of gut microbiota was measured using the abundance-based coverage estimator (ACE), Simpson, and Shannon indices (Figure 4). No significant variation was observed in the Shannon and Simpson indices among the four groups. However, the ACE index in the HPA group was significantly higher than in the NC and LPA groups. Principal Coordinate Analysis (PCoA) of binary-Jaccard indices demonstrated the diversity of bacteria among the four groups (Figure 5), with the HPA group showing distinct separation from the other three groups. The results of the microbiota in each group revealed that the relative abundance of the genus *Ligilactobacillus*, as well as the species *Lactobacillus agillis* and *Lactobacillus johnsonii*, in the PC group was significantly lower than in the NC group (*p* < 0.05, Figure 6; Figure 7). Additionally, the abundance of *Alistipes* and *Pedobacter* found in the HPA group was significantly higher than that in the PC group (*p* < 0.05, Figure 6). Moreover, the genus *Ligilactobacillus* in both the HPA and LPA groups was significantly lower compared to that in the NC group (*p* < 0.05).

## Discussion

*Cymbopogon martini* EO is an essential oil that has been proven to inhibit *C. albicans in vitro*, therefore, this study was designed to investigate the effect of *C*. *martini* EO on antioxidant activities, immune and intestinal barrier-related responses, and gut microbiota in *C. albicans*-infected pigeons.

Antioxidant enzymes are a class of enzymes that play an important role in the antioxidant activities of organisms. They protect cells and tissues from oxidative stress by catalyzing oxidative reactions and reducing or neutralizing harmful oxidative substances, including SOD, CAT, and GSH-Px. Studies have reported that pathogen infections could damage the antioxidant system and its functions (Ahmad, 2014). This study also found that *C. albicans* infection could reduce the levels of T-AOC, SOD, GSH-Px, and CAT in the serum of pigeons. *Candida albicans* infection can lead to an increased oxidative stress response in pigeons, resulting in a reduction in antioxidant activity. *Candida albicans* directly triggers oxidative stress by producing a series of metabolites and toxins, such as reactive oxygen species and peroxides. These reactive oxygen species can damage the pigeon’s antioxidant defense system, including SOD and GSH-Px, leading to a decline in antioxidant capacity (Yan et al., 2013). Previous studies have shown that the inhibition of antioxidant enzyme activities is detrimental to animal health (Baker et al., 2004), and essential oils can significantly enhance antioxidation to reduce oxidative stress in animals (Zhang et al., 2011; Abdel-Tawwab et al., 2018). In this study, the results showed that the levels of T-SOD and GSH-Px in the *C. martini* EO treatment groups were significantly increased compared to the *C. albicans* infected/untreated groups, especially the 2% *C*. *martini* EO -treated group. These results may be because *C*. *martini* EO has a high level of natural antioxidants (Sinha et al., 2011), which have the potential ability to scavenge free radicals, chelate transtegmental ions, and decompose peroxides (Embuscado, 2015). Similar studies have found that dietary essential oils significantly enhance the level of serum antioxidant activity (Rasheed et al., 2023).

Tight junction (TJ) proteins play a crucial role in the formation and maintenance of TJs, specialized intercellular structures that establish a seal between adjacent cells. They regulate the movement of molecules and pathogens across epithelial and endothelial cell layers (González-Mariscal et al., 2003). Claudins, Occludins, and ZOs are essential components of TJ proteins, responsible for maintaining the integrity and selective permeability of epithelial and endothelial barriers (Inai et al., 1999; Heiskala et al., 2001; Suzuki, 2020). The expression levels of *Claudin-1*, *Occludin*, and *Z O -1* genes can directly influence the abundance and localization of the corresponding proteins within the tight junctions. Decreased expression of these genes can result in reduced levels of their respective proteins in tight junctions, leading to compromised barrier integrity and increased permeability. In this study, we observed a significant decrease in the expression of *Claudin-1*, *Occludin*, and *Z O -1* in the *C. albicans* infected group compared to the uninfected group, except in crop tissue. This reduction suggests a potential disruption in the structural organization and function of TJs, leading to increased paracellular permeability and compromising the barrier’s ability to prevent the entry of pathogens. However, the expression of *Occludin* in infected pigeon crop tissues was higher than that in uninfected pigeon. This increase in *Occludin* expression could be attributed to the production of virulence factors or the secretion of molecules by *C. albicans*. These fungal factors could trigger signaling pathways in the host cells, leading to the increased expression of *Occludin* mRNA. In addition, our experimental results demonstrated a significant increase in the expressions of *Claudin-1*, *Occludin*, and *Z O -1* in the groups treated with *C*. *martini* EO, particularly at a concentration of 2%, compared to the *C. albicans* infected/untreated group. This elevation in *Claudin-1*, *Occludin*, and *Z O -1* expression indicates the potential restoration of TJ integrity. Potentially, *C*. *martini* EO treatment could contribute to the repair of the structural organization and function of TJs, thereby improving barrier integrity and reducing paracellular permeability to prevent pathogen entry.

For the immune-related genes, the results of this experiment demonstrated a significant increase in the expression of *IL-8*, *TGF-β*, and *IL-1*β in the *C. albicans* infected group, which is consistent with previous reports (Letterio et al., 2001; Schaller et al., 2002). Previous study reported that *C. albicans* releases various inflammatory factors during the infection, which can activate host immune cells and trigger an inflammatory response. Additionally, the cell wall components of *C. albicans* can stimulate the host immune system to produce an inflammatory response. The polysaccharide substances in the cell wall can interact with recognition receptors on the surface of host immune cells, triggering inflammatory signaling pathways and leading to inflammatory responses (Richardson and Moyes, 2015). These experimental results indicated that the treatment of *C*. *martini* EO significantly reduced the elevated levels of *IL-8*, *TGF-β*, and *IL-1*β caused by *C. albicans* infection. This suggests that *C. martini* EO treatment can alleviate inflammatory responses. Therefore, *C*. *martini* EO treatment is beneficial for the overall health recovery of *C. albicans*-infected pigeons.

It has been previously shown that *C. albicans* challenge or essential oils can alter the structure of the gut microbiota in pigeons (Tiihonen et al., 2010; Hu et al., 2021). However, according to the results of alpha and beta-indices, *C. albicans* and *C*. *martini* EO had no significant effect on pigeon intestinal bacterial composition in this study. Typically, microbial species such as *Lactobacillus agilis*, *Lactobacillus johnsonii,* and *Ligilactobacillus* are probiotics belonging to the *Lactobacillus* genus. They promote intestinal health, maintain the balance of intestinal flora, inhibit the growth of harmful bacteria, and promote the growth of beneficial bacteria (Fooks and Gibson, 2002; Reid and Burton, 2002). This experimental study found that *C. albicans* infection resulted in a decrease in *Lactobacillus* in the pigeon’s gut, and even 2% *C*. *martini* EO treatment did not improve this condition. However, this study found that *Alistipes* and *Pedobacter* in the 2% *C*. *martini* EO -treated group significantly increased. *Alistipes* and *Pedobacter* which have multiple functions and potential benefits in the gut. They are involved in carbohydrate metabolism, degrading and utilizing indigestible cellulose and other complex carbohydrates, thereby producing short-chain fatty acids (SCFA) such as propionic acid, butyric acid, and lactic acid Margesin and Shivaji, 2015). These SCFAs serve as the main energy source for intestinal cells and play an important role in gut health and function. They regulate the function of the intestinal immune system and maintain intestinal immune balance by interacting with host immune cells (Martin-Gallausiaux et al., 2021). Additionally, the *Pedobacter* strain can inhibit potentially pathogenic microorganisms. They can inhibit the growth and spread of pathogens by competing for resources, producing antibiotic-like substances, or creating unfavorable environmental conditions (Wong et al., 2011). Therefore, it could be inferred that 2% *C. martini* EO may potentially promote the growth of SCFA-producing bacteria and maintain intestinal immune balance, thereby aiding in the resistance of pigeons to *C. albicans*. According to the previous studies, *C. martini* EO can improve the antioxidant activity, immune response and intestinal function of animals. We hold the view that he restoration of antioxidant capacity, immune response, intestinal barrier function and intestinal microbiota in pigeons infected with *C. albicans* is attributed to the enhanced immune function that induced by *C. martini* EO (Andrade et al., 2014; Kiani et al., 2022).

## Conclusion

The use of 2% *C*. *martini* EO enhanced the antioxidant activity and the expression of genes related to intestinal barrier function but also inhibited inflammatory genes in *C. albicans*-infected pigeons. Additionally, 2% *C*. *martini* EO promoted the growth of SCFA-producing gut bacteria and helped maintain intestinal immune balance, thereby aiding in the resistance of pigeons to *C. albicans*. These results provide the basis for the development of an alternative to antibiotics for preventing *C. albicans* infection in pigeons.

## Data Availability

The datasets presented in this study can be found in online repositories. The names of the repository/repositories and accession number(s) can be found in the article/Supplementary Material.
