# Supplementary material for: Effect of Cymbopogon martini (Roxb.) Will.Watson essential oil on antioxidant activity, immune and intestinal barrier-related function, and gut microbiota in pigeons infected by Candida albicans
Source: Front Pharmacol. 2024 Apr 2;15:1380277. doi: 10.3389/fphar.2024.1380277 (PMC11018936; doi:10.3389/fphar.2024.1380277)
Supplement: Supplementary file 1 [file Table1.pdf]

Table S1 Composition of basal diet used in the experiment (air-dry basis, %).

Ingredient	Content	Nutritive index	Nutritive levels
Corn	40	Metabolisable energy (kcal/kg)	2785.6
Sorghum	10	Crude protein	15.2
Wheat	10	Calcium	1.42
Pea	10	Available phosphorus	0.53
Mung bean	10	Lysine	1.24
Soybean meal	13.5	Methionine	0.50
Soybean oil	1		
$CaHPO_4 \cdot 2H_2O$	2.8		
Salt	0.4		
Lysine	0.4		
Methionine	0.13		
Vitamins	0.05		
Minerals	0.15		
Choline chloride	0.07		
Sand	1.5		
Total	100		

Table S2 Primers used for qPCR

Target gene	Forward sequence (5’ to 3’)	Reverse sequence (5’ to 3’)
IL-1β	TGGCGTTTGTCCCTGATTTG	AGGCGGGGTTTCTTCTGC
TGF-β	ACTGAGACTGTGCGTGAGTG	AAGATGTCTCCGTTGGGCTG
Claudin-1	GCGGGGGACTACAGCTCTTG	AGTCGTACACCTTGCACTGG
IL-8	AGCTACTCTGAAGGACGGCA	ATCAGAATTGAGTTGAGCCTTGG
Occludin	TCGTCTGCGGGTTCCTCATA	CCACATTCTTCACCCACTCCT
ZO-1	CTGCCACACTGTGACCCC	AGTGGTCAATTAGGACTGAGACA
β-actin	GAGAAATAGTGCGTGACATCA	CCTGAACCTCTCATTGCCA
